# Colchicine efficacy comparison at varying time points in the peri-operative period for coronary artery disease: a systematic review and meta-analysis of randomized controlled trials

**DOI:** 10.3389/fcvm.2023.1156980

**Published:** 2023-08-04

**Authors:** Zhi-Yang Wei, Jun-Yu Lai, Ya-Ting Li, Xiao-Yan Yu, Yan-Hong Liu, Jing-Xuan Hu, Bei-Bei Gao, Jian-Guang Wu

**Affiliations:** ^1^ Department of Postgraduate, Jiangxi University of Traditional Chinese Medicine, Nanchang, China; ^2^ Cardiology Department, Affiliated Hospital of Jiangxi University of Traditional Chinese Medicine, Nanchang, China

**Keywords:** colchicine, coronary artery disease, percutaneous coronary intervention, intervention time point, systematic review, meta-analysis

## Abstract

**Objectives:**

Over the years, it has been found that colchicine offers substantial benefits in secondary prevention in patients with coronary artery disease (CAD). We studied the effects of colchicine timing because there are no guidelines about when to provide it during the perioperative period for patients with CAD.

**Methods:**

Up to January 1, 2023, seven electronic literature databases were screened (including three English databases and four Chinese databases). Randomized controlled trials included only treatment with colchicine in the perioperative period of CAD. The Cochrane Evaluation Tool was used to judge the risk of bias in research. Statistical analysis was performed by Stata 16.0 software.

**Results:**

We evaluated twelve studies that found colchicine to be effective in decreasing the occurrence of major adverse cardiac events (MACEs) (*p* < 0.00001), but it also raised the rate of adverse events (*p* = 0.001). Subgroup analysis showed the same benefit in lowering the incidence of MACE with continuous administration of a total daily dose of 0.5 mg postoperatively while minimizing drug-related side effects in the patients (*p* = 0.03). When it comes to preventing surgical stroke occurrences, postoperative administration is more effective (*p* = 0.006). While the effect of simultaneous preoperative and postoperative administration was marginally greater than other periods in reducing postoperative hs-CRP levels (*p* = 0.02).

**Conclusion:**

Colchicine, a traditional anti-inflammatory drug, also reduces the risk of MACE by reducing inflammation after PCI. Administration at different periods had no significant effect on decreasing the occurrence of MACE, but when administered postoperatively, we advise continuous administration with a total daily dose of 0.5 mg to obtain the same benefit while minimizing the drug's side effects. Postoperative administration is the better measure to prevent postoperative stroke events. Due to the effective anti-inflammatory effect of colchicine, we recommend its use as early as possible in the perioperative period and its continued use at low doses in the postoperative period.

**Systematic Review Registration:**

https://www.crd.york.ac.uk/PROSPERO/display_record.php?RecordID=316751, identifier CRD42022316751.

## 1. Introduction

Coronary artery disease (CAD) is the leading cause of morbidity and mortality worldwide, and population growth and aging have contributed to a rise in the number of cardiovascular deaths in recent years (1). The European Society of Cardiology suggested that patients with CAD change their lifestyles by quitting smoking, engaging in regular exercise, eating healthier, and controlling their underlying diseases like hypertension, diabetes, and hyperlipidemia., they also recommend taking regular antithrombotic treatment (2–4). When necessary, surgical procedures such as percutaneous coronary intervention (PCI) and coronary artery bypass graft (CABG) are undertaken (5). The entire process of developing CAD involves inflammation, increases intravascular plaque instability (6), which causes intravascular damage after PCI, which aggravates the inflammatory response and sets the scene for intravascular plaque rupture or erosion and myocardial injury and infarction (7, 8). As shown in a study, the sensitive systemic inflammatory indicator hs-CRP independently predicts the risk of coronary events (9), as an inflammatory factor with increased production in patients with myocardial infarction, high levels of IL-6 also tend to predict a range of other coronary events such as poorer prognosis (10, 11). As a result, decreasing inflammation after myocardial infarction may improve prognosis (12). Since PCI aims to decrease the occurrence of postoperative cardiovascular events, inflammation is not only a major contributor to their development but may also be connected to an increased risk of mortality in postoperative patients, which constrains this treatment strategy (13, 14). This shows that the current treatment methods still have space for improvement.

Colchicine is a conventional and inexpensive anti-inflammatory medication that is frequently known for therapy for acute gout and other inflammatory conditions including pericarditis (15). It mainly inhibits microtubule aggregation, inhibits cell mobility, adhesion, and activation in immune cells, and exerts anti-inflammatory effects through inhibition of the inflammasome pathway (16). Perioperative inflammation has been related to major adverse cardiac events (MACEs), which may be identified an hour after PCI, according to earlier studies (17, 18). When administered acutely before PCI to patients with the acute coronary syndrome (ACS), colchicine decreases the synthesis of local cardiac inflammatory cytokines (19); the COLCOT trial revealed that early administration after myocardial infarction was more effective in lowering the risk of ischemic cardiovascular events (20), these may result from the benefits associated with administration at different times. Therefore, although many studies have found various benefits of colchicine for patients with CAD, these studies have not yet addressed the ideal time point for colchicine treatment during the PCI perioperative period, and the time point for colchicine administration has not been systematically evaluated. To evaluate the impact of colchicine at various time points in the perioperative period of PCI in a randomized controlled trial, we conducted a systematic evaluation and meta-analysis.

## 2. Materials and methods

This study's protocol has been published in PROSPERO (registration number: CRD42022316751). The Preferred Reporting Items for Systematic Reviews and Meta-Analyses (PRISMA) guidelines were followed for undertaking this research.

### 2.1. Data source and search strategy

The clinical RCTs of colchicine with PCI for CAD were searched in the relevant database, including the Chinese VIP database (VIP), China National Knowledge Infrastructure Database (CNKI), WanFang Medical database, and Chinese Biomedical Database (CBM), and three English databases (PubMed, Embase, Cochrane Library). The retrieval dates were from inception dates to January 1, 2023. The search was performed using the strategy of combining MeSH terms with free text search terms, MeSH terms including CAD, ACS, PCI, and colchicine, with no restrictions on language or publishing status. The search process is described in Supplementary Text S1.

### 2.2. Inclusion and exclusion criteria

① Participants: aged >18 years who underwent PCI in compliance with local guidelines for the treatment of coronary artery disease.
② Interventions and Comparisons: The study population was separated into two groups: the experimental and the control. During the PCI peri-procedure period, the experimental group received colchicine or colchicine in conjunction with conventional therapy, whereas the control group received a placebo or conventional antithrombotic and anticoagulant therapy. The period leading up to surgery, including the time before, during, and after PCI, defined as the perioperative period. It starts when a patient decides to have surgery and lasts until basic recovery.
③ Outcomes
  • Primary outcomes: MACEs, mainly including stent thrombosis, myocardial infarction (MI), stroke, in-stent restenosis (ISR), cardiac arrest, and all-cause death.
④ Secondary outcomes:
  • Secondary outcomes: all-cause mortality; ISR; MI; stroke; stent thrombosis; inflammatory response markers such as hs-CRP and IL-6; Because postoperative adverse events (mostly gastrointestinal symptoms, allergic reactions, etc.) are typical side effects of colchicine, we were interested in determining if the administration of the treatment at different periods changed.
Types of Studies: Clinical randomized controlled trials that are not constrained by time, language, or whether they are blinding or not.

### 2.3. Exclusion criteria

• The timing of perioperative dosing for PCI is not indicated
• No corresponding outcome indicator
• Statistical results are wrong or data are repeated
• The article has only an abstract but no full text or the full text is not available
• Non-randomized controlled trials

### 2.4. Data extraction and quality assessment

The select results were imported into Endnote20 software for management. Extracted data included basic information about the study (first authors, publication year, study design, etc.), participants' characteristics (average age, sex composition, sample size, etc.), interventions (experiment group interventions, control group interventions, drug intervention times, doses), outcomes (All-cause death, ISR, inflammatory response markers, etc.). Reasons for the exclusion of relevant information were noted during the screening process to facilitate review and further evaluation. When there was a difference of opinion throughout the screening procedure, a third researcher was consulted to reach a judgment.

To evaluate the risk of bias in the included RCTs, the Cochrane Collaboration's tool was utilized, according to the following criteria: ① random sequence generation; ② allocation concealment; ③ blinding participants and personnel; ④ blinding outcome

assessment; ⑤ incomplete outcome data; ⑥ selective reporting; ⑦ other bias. A third researcher was consulted when evaluating literature where it was difficult to assess quality.

### 2.5. Data synthesis

Meta-analysis was conducted by using Stata v 16.0 software (StataCorp, College Station, TX, United States of America). The heterogeneity of each study was analyzed using the chi-square test. If *I*^2 ^≤^ ^50%, *p* ≥ 0.1, indicating that the heterogeneity between multiple studies was marginal, for meta-analysis, the fixed-effect model was applied. If *I*^2 ^>^ ^50%, *p*^ ^<^ ^0.1, indicating that the heterogeneity between studies is large, for meta-analysis, the random effect model was applied, and the chi-square test was used for heterogeneity analysis. Continuous variables are given as the standardized mean difference (SMD) with 95% confidence intervals (CIs), while count data are provided as relative risk (RR) or odds ratio (OR) with 95% CIs. The statistical heterogeneity between the results of the studies was used for the analysis of the causes of heterogeneity, using subgroup analysis and sensitivity analysis.

## 3. Results

### 3.1. Literature screening result

**Figure 1:**
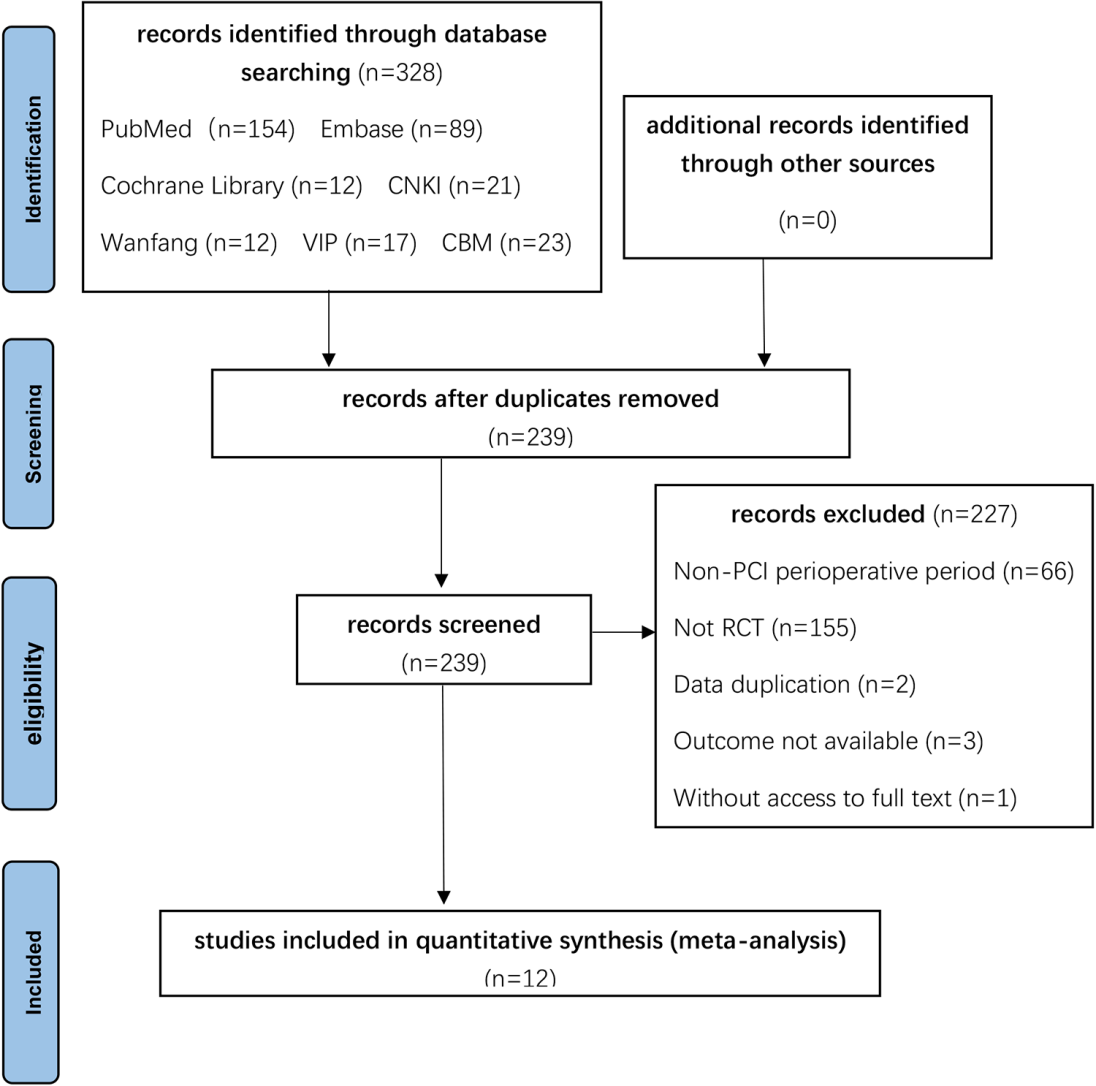
Process of study extracted for the meta-analysis.

239 articles were discovered and reviewed after duplicate research articles were removed. By the PICOS principle, we eliminated 66 studies that only received oral colchicine treatment without PCI, 155 non-randomized controlled trials, 2 studies with repetitive data, 3 studies with inconsistent observational indicators, and 1 study without full text. Finally, 12 randomized controlled studies were included. The specific search process can be found in Figure 1.

### 3.2. Basic characteristics of the included study

The samples for the 12 studies contained a total of 7,591 patients, among them, there were 3,862 in the control group and 3,729 in the experimental group. The ages of the patients varied from 47 to 77. The age, gender, and underlying diseases of the two groups' baseline data were mostly comparable and consistent. Two of these studies used colchicine preoperatively (21, 22); Five studies used colchicine in the postoperative period (23–27); Five studies used colchicine both pre-and post-operatively for PCI (28–32). details are shown in Tables 1, 2.

**Table 1:** Main characteristics of the included studies.

Study (author/year)	Sample size (T/C)	Age (years) (T/C)	Male (%)	Drug intervention time	Dosing time	Intervention (T/C)		Main outcomes	PCI (%)
Shah et al. (22)	400 (206/194)	59/62	93.5	Preoperative	2 h	Colchicine (1.2 mg + 0.6 mg)	placebo	①②④⑤⑥⑦⑧	100
cole et al. (21)	75 (36/39)	63.7 ± 6.9/63.5 ± 7.2	72.0	Preoperative	6–24 h	Colchicine (1 mg + 0.5mg)	placebo	④	100
Akodad et al. (23)	44 (23/21)	60.1 ± 13.1/59.7 ± 11.4	79.5	Postoperative	1 month	Colchicine (1 mg qd)	placebo	①⑥⑧	100
Tardif et al. (26)	4,745 (2,366/2,379)	60.6 ± 10.7/60.6 ± 10.7	80.8	Postoperative	22.6 months	Colchicine (0.5 mg qd)	placebo	①②④⑥⑦ ⑧	92.9
Tong et al. (27)	795 (396/399)	59.7 ± 10.2/60.0 ± 10.4	79.4	Postoperative	12 months	Colchicine (0.5 mg bid + 0.5 mg qd)	placebo	①②③⑥⑦	86.9
Deftereos et al. (24)	196 (100/96)	63.7 ± 6.9/63.5 ± 7.2	65.3	Postoperative	6 months	Colchicine (0.5 mg bid)	placebo	①②⑥⑦⑨	100
Hennessy et al. (25)	237 (119/118)	61 ± 13.6/61 ± 12.5	76.7	Postoperative	1 month	Colchicine (0.5 mg qd)	placebo	①③④⑤⑥⑧	100
O'Keefe et al. (31)	197 (130/67)	59/62	85.7	Preoperative postoperative	6 months	Colchicine (0.6 mg bid)	placebo	①⑥⑦⑨	100
Mewton et al. (30)	192 (101/91)	59.0 ± 10.6/60.9 ± 10.4	80.2	Preoperative postoperative	5 days	Colchicine (2 mg + 0.5 mg bid)	placebo	①②④⑥	100
Zarpelon et al. (32)	140 (71/69)	61.5 ± 10.3/60.3 ± 8.1	67.8	Preoperative postoperative	<1 month	Colchicine (1 mg bid + 0.5 mg bid)	placebo	①⑦	100
Akrami et al. (28)	249 (120/129)	56.9 ± 7.56/56.89 ± 7.45	69.4	Preoperative postoperative	6 months	Colchicine (0.5 mg qd)	placebo	①⑦	86.3
Hosseini et al. (29)	321 (120/129)	58.7 ± 10.4/58.9 ± 11.2	79.1	Preoperative postoperative	12 months	Colchicine (1 mg + 0.5 mg qd)	Placebo	①	100

T, trial group; C, control group; qd, once a day; bid, twice a day. ① MACEs; ② stroke; ③ stent thrombosis; ④ hs-CRP; ⑤ IL-6; ⑥ adverse events; ⑦mortality; ⑧ MI; ⑨ ISR.

**Table 2:** Key features of included studys.

Study (author/year)	Key inclusion criteria	Key exclusion criteria	Time of colchicineinitiation and duration	Dosage
Shah et al. (22)	suspected ischemic heart disease or acute coronary syndromes referred for clinically indicated coronary angiography with possible PCI	Glomerular filtration rate <30 ml/min or on dialysis and intolerance to colchicine	First dose 1–2 h before coronary angiography, second dose one hour later	1.2 mg 1 to 2 h before coronary angiography, followed by 0.6 mg 1 h later
cole et al. (21)	patients were included if they had a de-novo lesion amenable to PCI, and high-sensitive troponin-I and CKhad peaked and stabilized	Patients were excluded if they had active inflammation/infection;had prior ACS within 12months;had severe renal impairment	Medication given 6–24 h before PCI procedure	1 mg followed by 0.5 mg one hour later
Akodad et al. (23)	The patient with ST segment elevation myocardial infarction underwent successful primary percutaneous coronary intervention	Cardiogenic shock, severe chronic kidney failure, colchicine intolerance or contraindication	colchicine was administered on the first day of the surgery and for 1 month, without a loading dose	1 mg once daily
Tardif et al. (26)	Patients who had an MI within30 days before enrolment andhad completed any plannedpercutaneous revascularisationprocedures	Patients if had stroke within previous 3 months, type two index MI, recent or planned CABG; inflammatory bowel disease or chronic diarrhea and severe renal disease	Once the patient grouping is completed, medication administration begins, with a median treatment duration of 22.6 months	0.5 mg once daily
Tong et al. (27)	ACS with presence of coronary disease	Requiring bypass surgery; severe liver impairment; severe renal impairment (eGFR <30 ml/min/1.73 m^2^)	Patients start taking medication immediately after being assigned to a group, and the treatment duration is 12 months	0.5 mg oral colchicine twice daily for the first month, followed by 0.5 mg daily for eleven months
Deftereos et al. (24)	Diabetes and undergoing percutaneous coronary revascularization	Acute myocardial infarction; renal impairment (eGFR <20 ml/min/1.73 m^2^);liver failure	The patient began taking the medication on the day after PCI and continued for 6 months	0.5 mg twice daily
Hennessy et al. (25)	Adult patients were eligible for enrolment if they had sustained a type 1 acute MI	Severe renal impairment; severe hepatic dysfunction; females of child-bearing age who are pregnant, lactating	Dosing started after completion of grouping and continued for one month	0.5 mg once daily
O'Keefe et al. (31)	Patients (CCS) who had undergonesuccessful coronary angioplasty	Premenopausal women; active peptic ulcer disease and diarrhea; creatinine ≥2.5 mg/dI at baseline; known colchicine intolerance	The first dose should be administered within 24 h before or after the surgery, and the treatment should continue for a duration of six months	0.6 mg twice daily
Mewton et al. (30)	All adult patients with a first-time STEMI referred for primary or rescue PCI admitted to the participating centers were screened against eligibility criteria	Hemodynamic instability; any obvious contraindication to cardiac magnetic resonance imaging; severe liver or known renal dysfunction as defined by a glomerular filtration rate ≤30 ml/min and chronic treatment with colchicine	The preoperative administration of a loading dose of PCI was followed by postoperative medication for a duration of five days	2 mg oral loading dose, followed by 0.5 mg twice a day
Zarpelon et al. (32)	indication for electivemyocardial revascularization surgery	Severe liver disease and renal failure; known gastrointestinal diseases	Treatment group started preoperative dosing until discharge	1 mg given twice a day within 24 h before surgery, 0.5 mg twice a day after surgery
Akrami et al. (28)	All the patients underwent coronary angiography and were managed with either PCI or medical therapy	Any history of long-term colchicine use or hypersensitivity to it, moderate renal dysfunction (glomerular fltration rate >50)	Dosing started after completion of grouping and continued for six months	0.5 mg once daily
Hosseini et al. (29)	The patient was diagnosed with acute STEMI and underwent PCI within 12 h	Cardiogenic shock; colchicineintolerance; renal failure (estimated glomerular filtration rate, 30 ml/min)	Administered immediately before PCI in patients with STEMI; continuous postoperative dosing for one year	1 mg given preoperatively, followed by another 0.5 mg administeredorally each day

CK, creatinine kinase; eGFR, estimated glomerular filtration rate; PCI, percutaneous coronary intervention; ACS, acute coronary syndrome; CCS, chronic coronary syndrome; MI, myocardial infarction; STEMI, ST-segment elevation myocardial infarction; CABG, coronary artery bypass graft.

### 3.3. Specific interventions

In the two preoperative studies, the experimental group interventions were separated into two therapeutic doses: 1.2 mg given 1–2 h before PCI and 0.6 mg given immediately (22), and 1 mg given 6–24 h before PCI and 0.5 mg administered an hour after the surgery (21); in the five postoperative studies, the experimental group interventions: ① 0.5 mg qd (25, 26), ② 0.5 mg bid (24), ③ 1 mg qd (23), ④ 0.5 mg bid in the first month and 0.5 mg qd in the next 11 months after the operation (27); five studies used it both in pre-and postoperatively: ① 0.5 mg qd (28), ② 0.6 mg bid (31), ③ preoperative 1 mg, postoperative 0.5 mg qd (29), ④ preoperative 2 mg, postoperative 0.5 mg bid (30), ⑤preoperative 1 mg bid, postoperative 0.5 mg bid (32). All of the control groups received a placebo treatment. details are shown in Tables 1, 2.

### 3.4. Outcome indicators

The details are described in Table 1. only one preoperative study reported recurrent myocardial infarction, stroke, IL-6, adverse events, and mortality events among all the included indicators, two preoperative studies evaluated hs-CRP; five postoperative studies reported adverse events, three studies reported stroke, mortality, and myocardial infarction, two studies reported stent thrombosis, and two studies examined hs-CRP, but only one analyzed IL-6 and ISR; mortality events occurred in three pre-and postoperatively studies, two reported adverse events, only one assessed stroke, and hs-CRP, and one reported ISR.

### 3.5. Risk of bias

**Figure 2:**
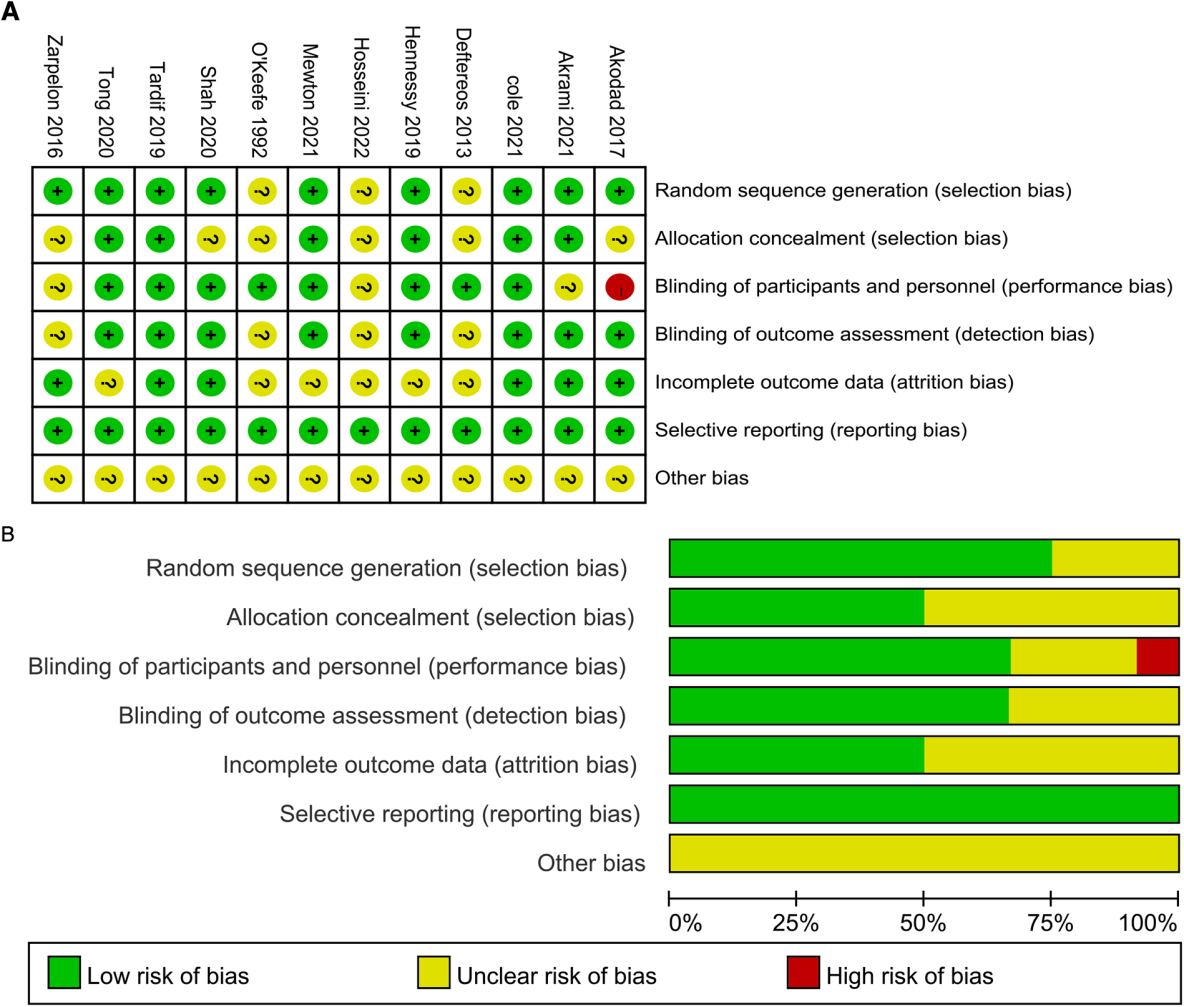
(**A**) Risk of bias summary. (**B**) Risk of bias graph.

Ten studies from twelve articles received a low risk of bias rating as a result of a simple randomization technique utilizing computer randomization and stratified group randomization (21–23, 25–30, 32), the remaining 2 studies only described randomization and did not illustrate the method (24, 31); five studies did not specify allocation concealment (22–24, 31, 32); we concluded that one study did not describe whether blinding was performed and judged this to be high risk (23); five studies failed to complete follow-up due to side effects (24, 25, 27, 30, 31); all studies did not involve selective reporting; other biases are unclear (Figure 2).

### 3.6. Results of the meta-analysis

#### 3.6.1. MACEs

**Figure 3:**
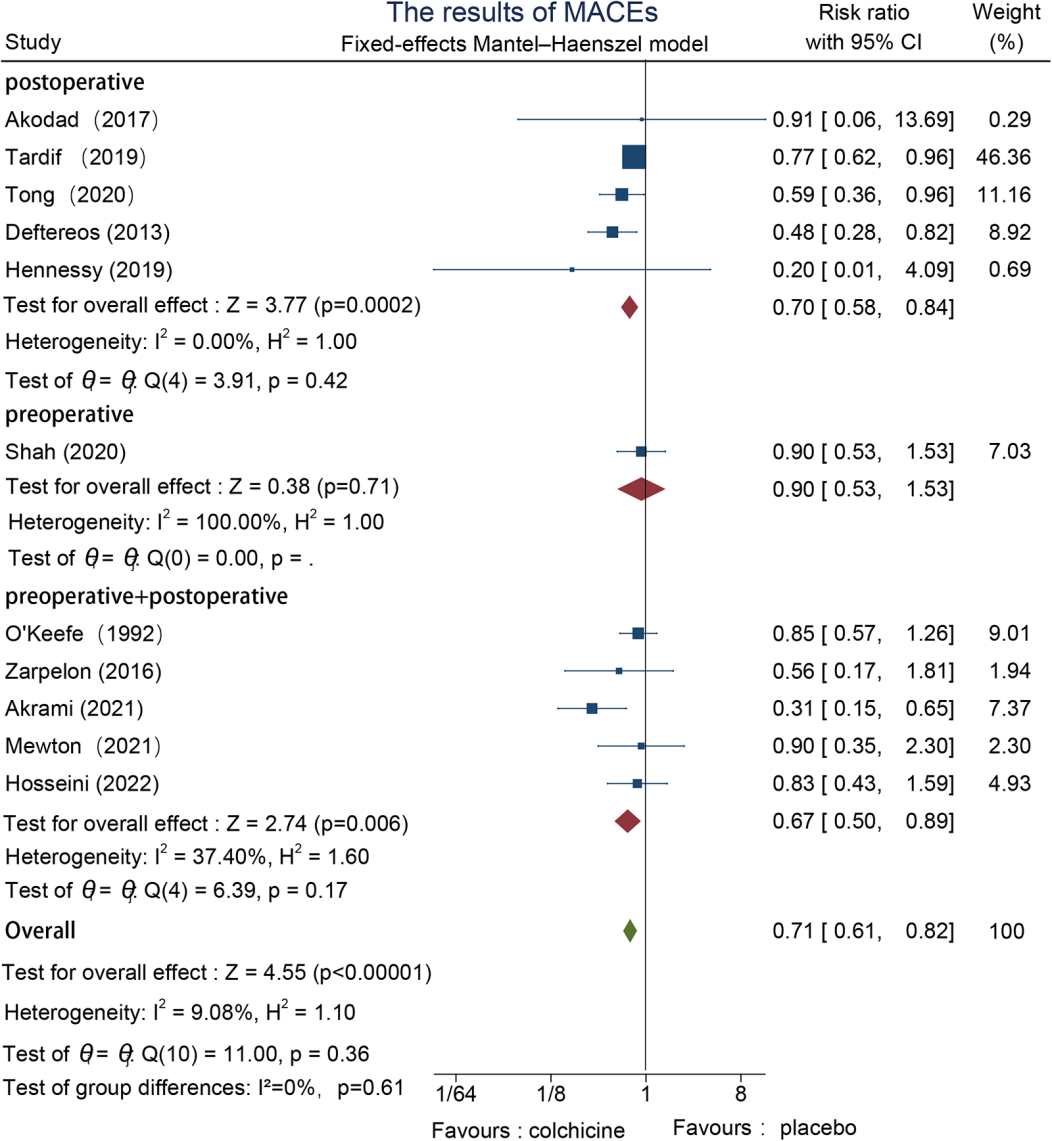
The results of MACEs.

MACEs were reported in 11 studies, one preoperatively (22), five postoperatively (23–27), and five both pre-and postoperatively (28–32). We used a fixed-effects model since the results had no significant heterogeneity. In conclusion, the incidence of MACEs was lower in the colchicine group (RR = 0.71, 95% CI [0.61, 0.82], *p* < 0.00001). Subgroup analysis showed that the pre-and postoperative group (RR = 0.67, 95% CI [0.50, 0.89], *p* = 0.006) was slightly lower than the postoperative group (RR = 0.70, 95% CI [0.58, 0.84], *p* = 0.0002). However, the difference between the postoperative intervention group and the pre-and postoperative intervention group was not significant (*I*^2 ^=^ ^0%, *p* = 0.61) (Figure 3).

#### 3.6.2. Stroke

**Figure 4:**
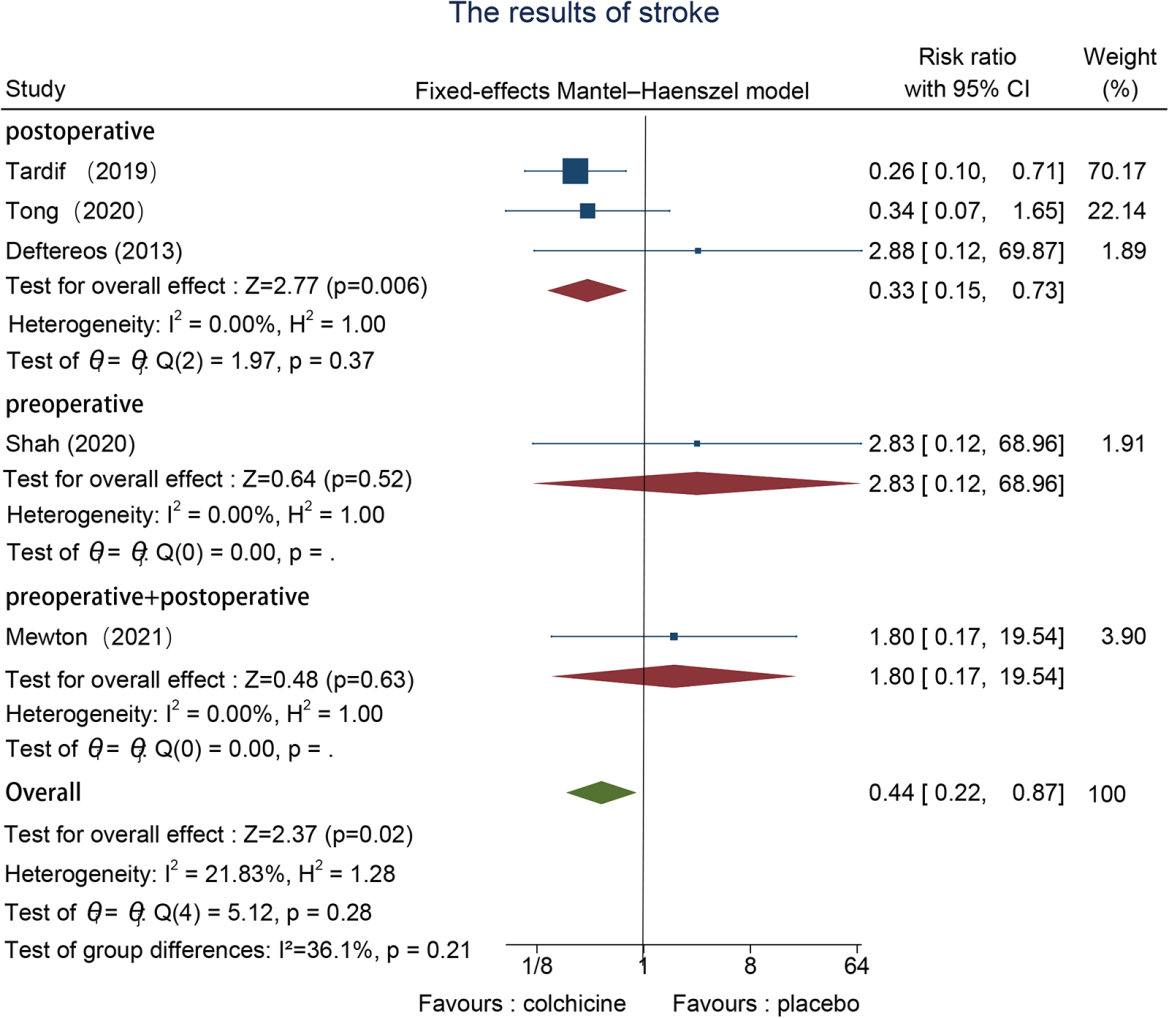
The results of stroke.

Five studies reported stroke events, one preoperative (22), three postoperative (24, 26, 27), and one in the pre-and postoperative group (30). There was no significant heterogeneity in the data results, which were assessed using a fixed effects model. overall, the stroke incidence in the colchicine group was markedly lower in contrast to the control group [RR = 0.44, 95% CI (0.22, 0.87), *p* = 0.02]. The stroke rate was lower in the postoperative intervention group, according to subgroup analysis [RR = 0.33, 95% CI (0.15, 0.73), *p* = 0.006] (Figure 4).

#### 3.6.3. Stent thrombosis

**Figure 5:**
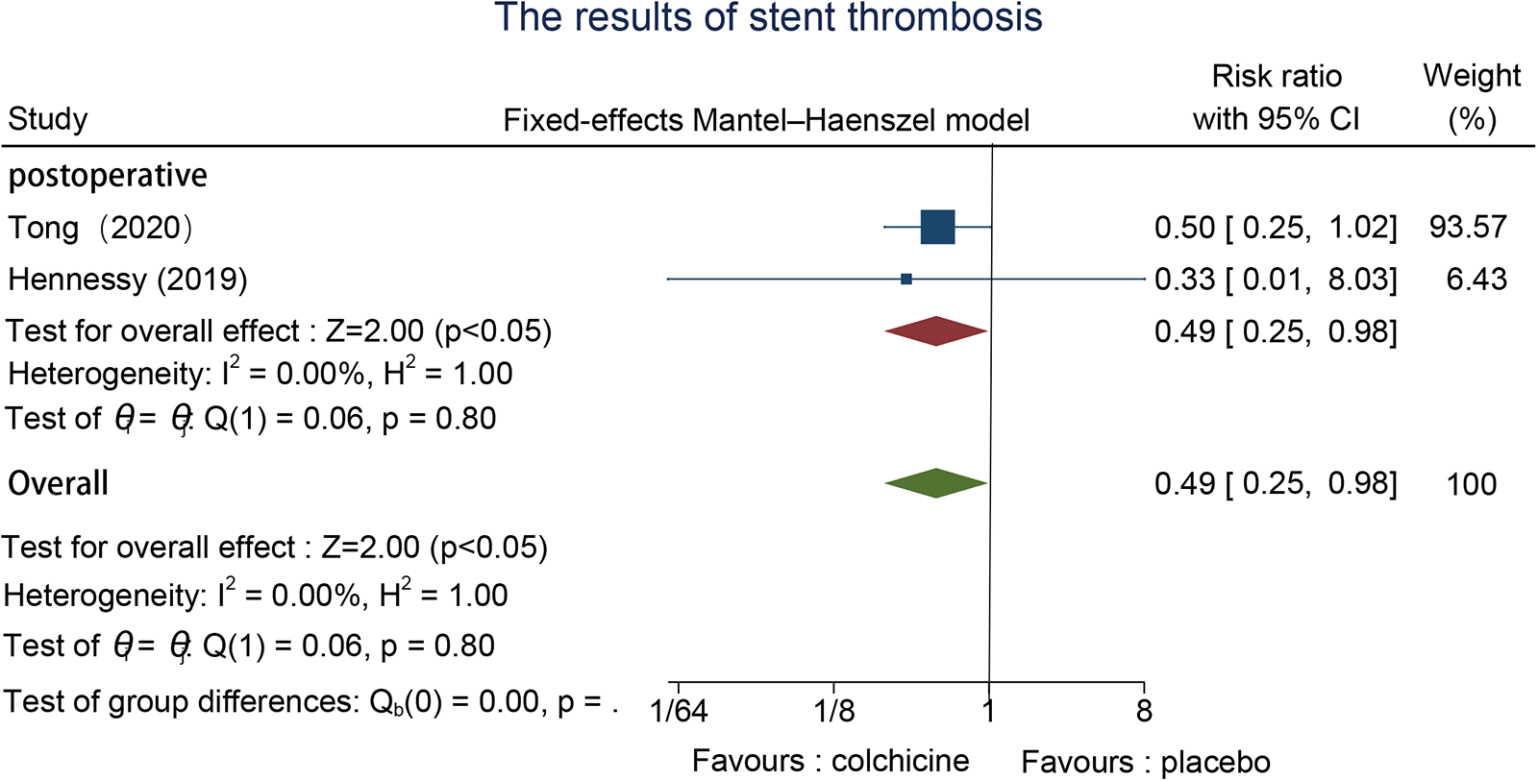
The results of stent thrombosis.

The effect of the timing of this colchicine use cannot be assessed yet, as the effect on stent thrombosis has only been reported in postoperative studies. Two studies evaluated stent thrombosis events (25, 27), we used a fixed effects model because of the low between-group heterogeneity. Colchicine can significantly decrease the incidence of stent thrombosis when in contrast to the control group [RR = 0.49, 95% CI (0.25, 0.98), *p* < 0.05] (Figure 5).

#### 3.6.4. Inflammatory response markers

**Figure 6:**
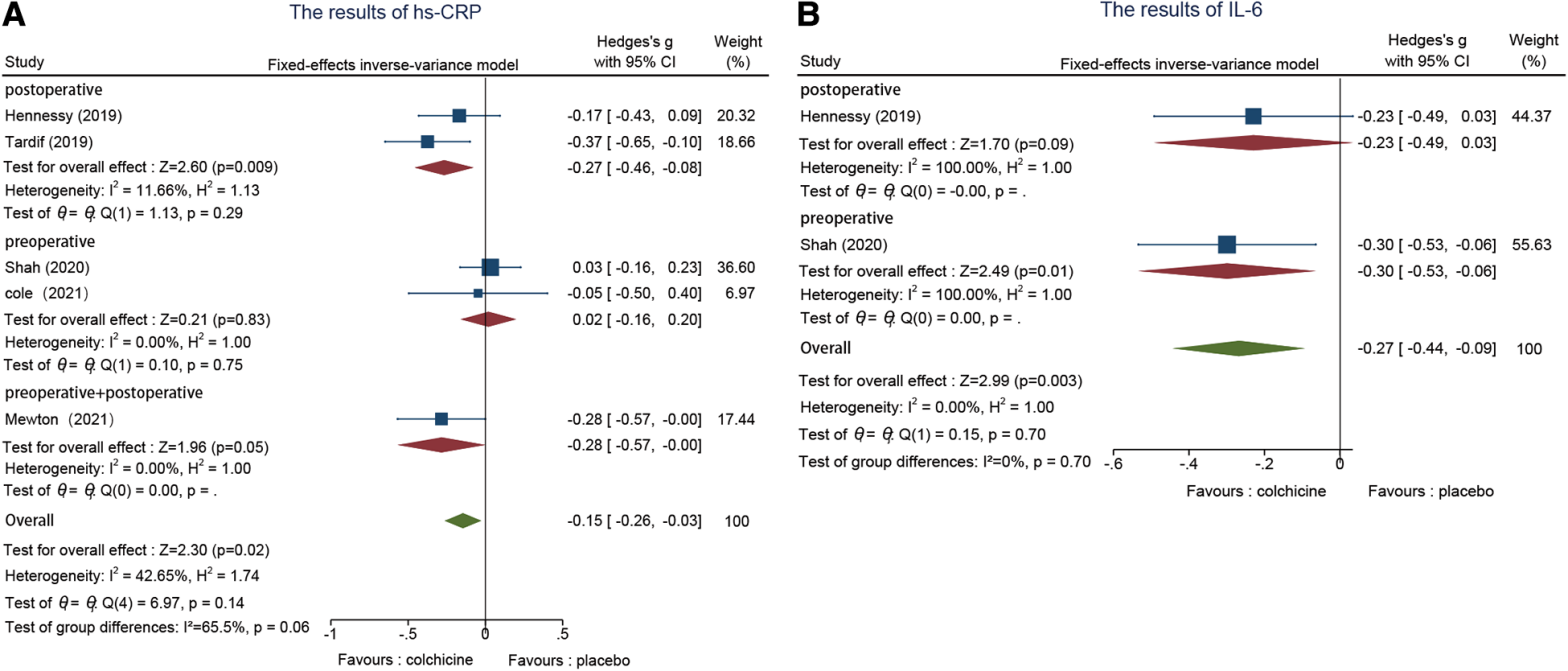
(**A**) The results of Hs-CRP. (**B**) The results of IL-6.

hs-CRP, five studies reported this indicator (21, 22, 25, 26, 30). We used the fixed effect model to analyze and revealed that the colchicine group reduced hs-CRP after PCI better than the control group (SMD = −0.15, 95% CI [−0.26, −0.03], *p* = 0.02). The outcomes of the meta-analysis demonstrate that the pre-and postoperative intervention groups (SMD = −0.27, 95% CI [−0.46, −0.08], *p* = 0.009) were slightly better than the postoperative intervention group (SMD = −0.28, 95% CI [−0.57, 0.00], *p* = 0.05), a significant difference between subgroups (Figure 6A).

IL-6, two studies analyzed IL-6 levels (22, 25). Colchicine was more effective than placebo treatment in decreasing IL-6 levels after PCI when compared to the control group, according to the fixed effects model (SMD = −0.27, 95% CI [−0.44, −0.09], *p* = 0.003). The results of the subgroup analysis indicated that the preoperative group was excellent to the postoperative group (Figure 6B).

#### 3.6.5. Adverse events

**Figure 7:**
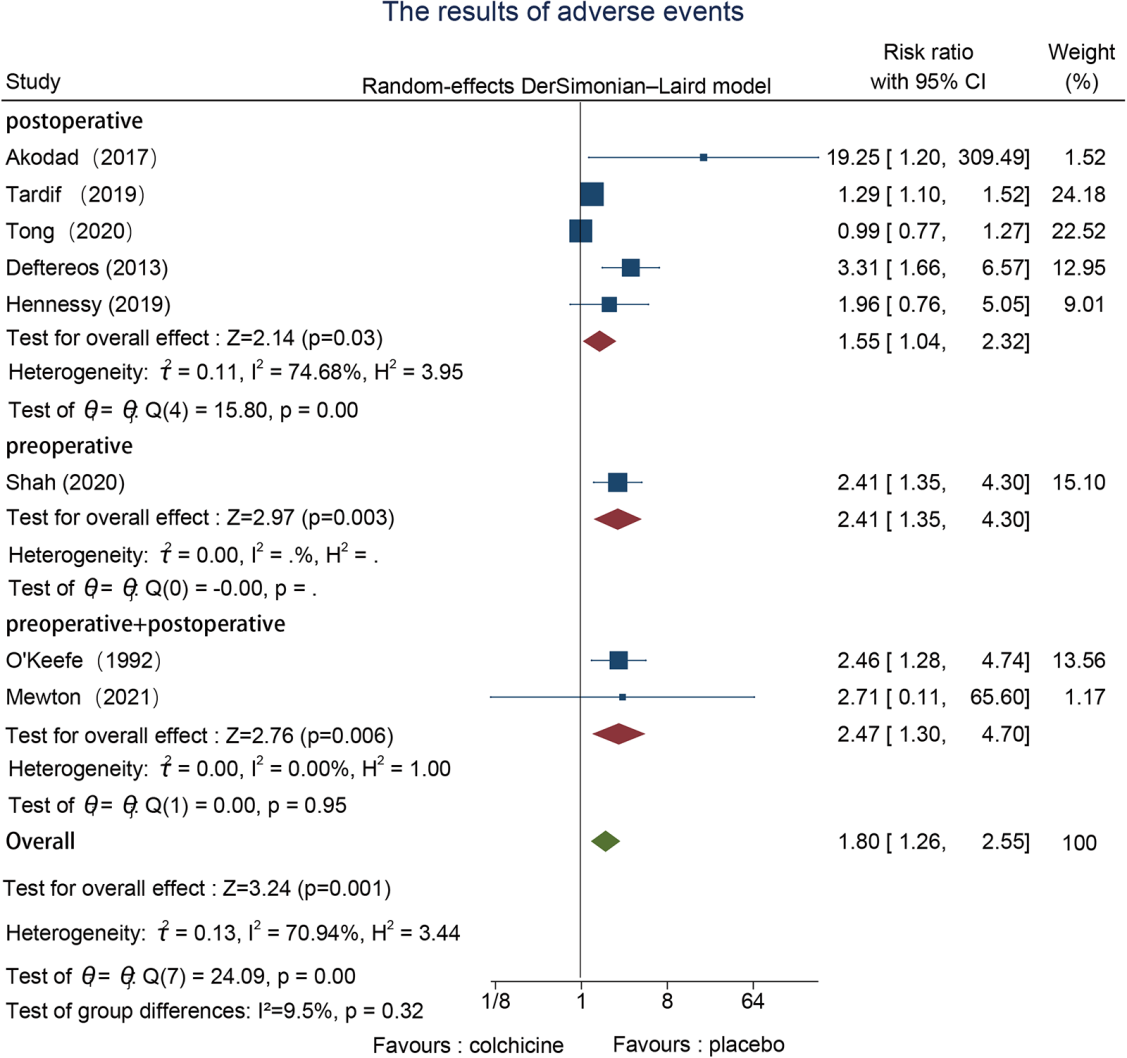
The results of adverse events.

Eight studies reported adverse events, one in the preoperative (22), and five in the postoperative (23–27), and two in both the pre-and postoperative groups (30, 31). We adopted a random effects model to evaluate and conduct subgroup analysis based on the time point of colchicine administration since we discovered that there was significant heterogeneity between groups. The findings of the meta-analysis indicated that heterogeneity was mainly concentrated in the postoperative group; the postoperative group (RR = 1.55, 95% CI [1.04, 2.32], *p* = 0.03) had a lower percentage of drug side effects than the other groups. However, the differences between subgroups were not significant (Figure 7).

#### 3.6.6. Mortality

**Figure 8:**
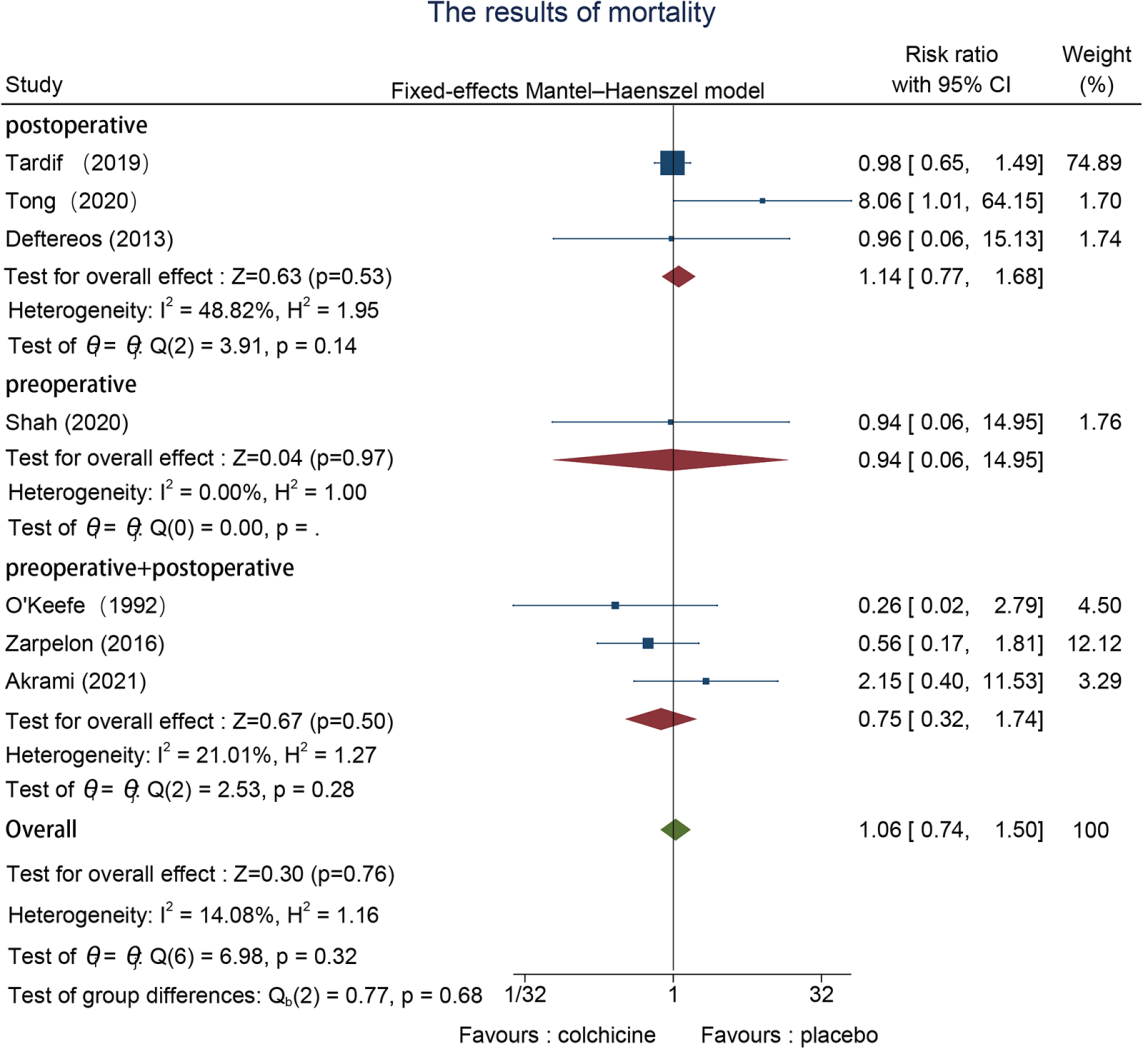
The results of mortality.

The effects on all-cause mortality were reported in one pre- (22), three using both pre- and post- (28, 31, 32), and three post-operative studies (24, 26, 27). Heterogeneity was not significant, so we used a fixed effects model. The outcome revealed no important difference in all-cause mortality with colchicine used in contrast to the control group (RR = 1.06, 95% CI [0.74, 1.50], *p* = 0.76) (Figure 8).

#### 3.6.7. MI

**Figure 9:**
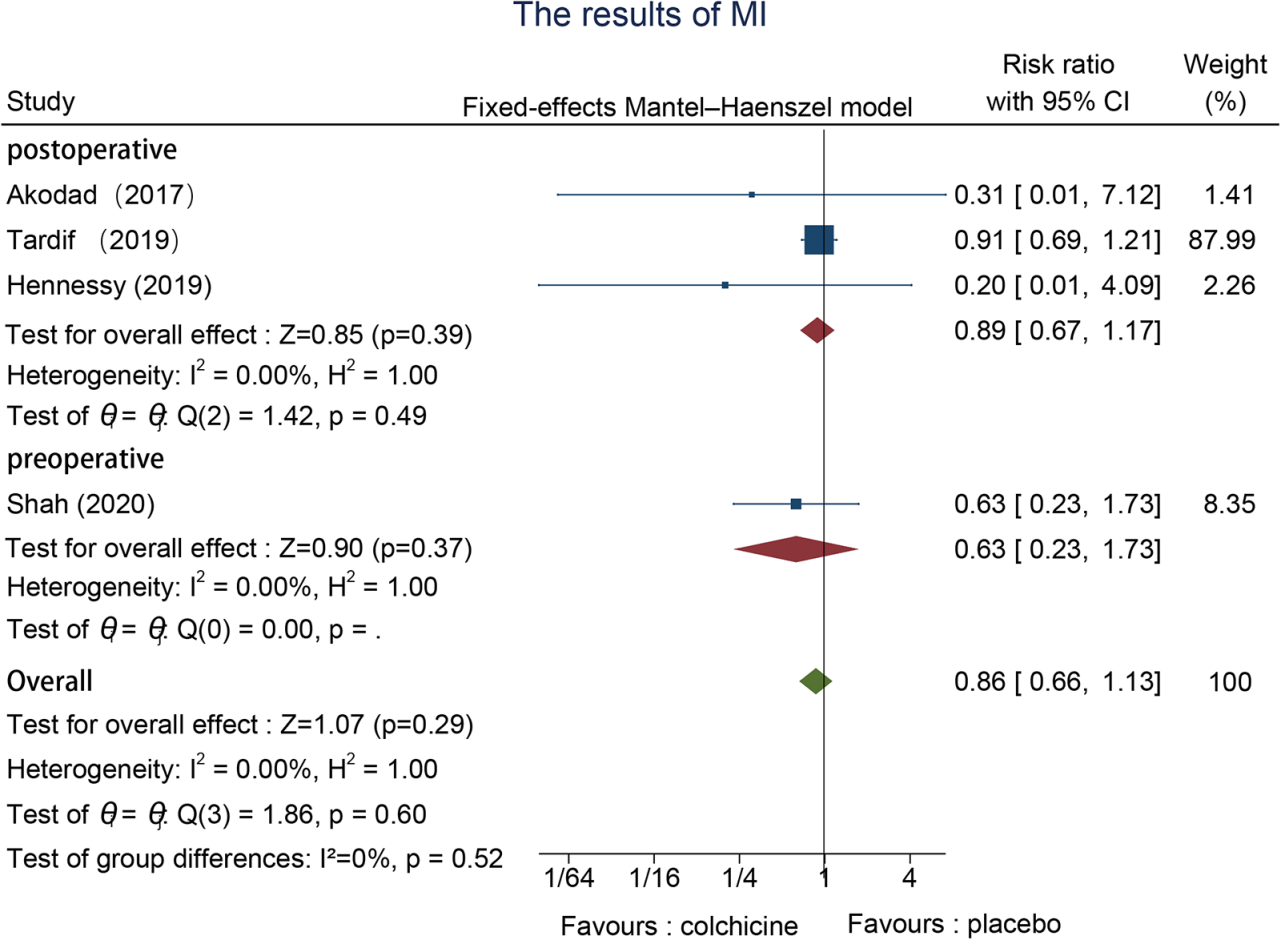
The results of MI.

Four studies recorded MI events, one preoperative (22) and three postoperative (23, 25, 26), with no remarkable heterogeneity in outcomes, which we adopted a fixed-effects model. The effectiveness of colchicine use on MI was not statistically relevant in contrast to the control group (RR = 0.86, 95% CI [0.66, 1.13], *p* = 0.29) (Figure 9).

#### 3.6.8. ISR

**Figure 10:**
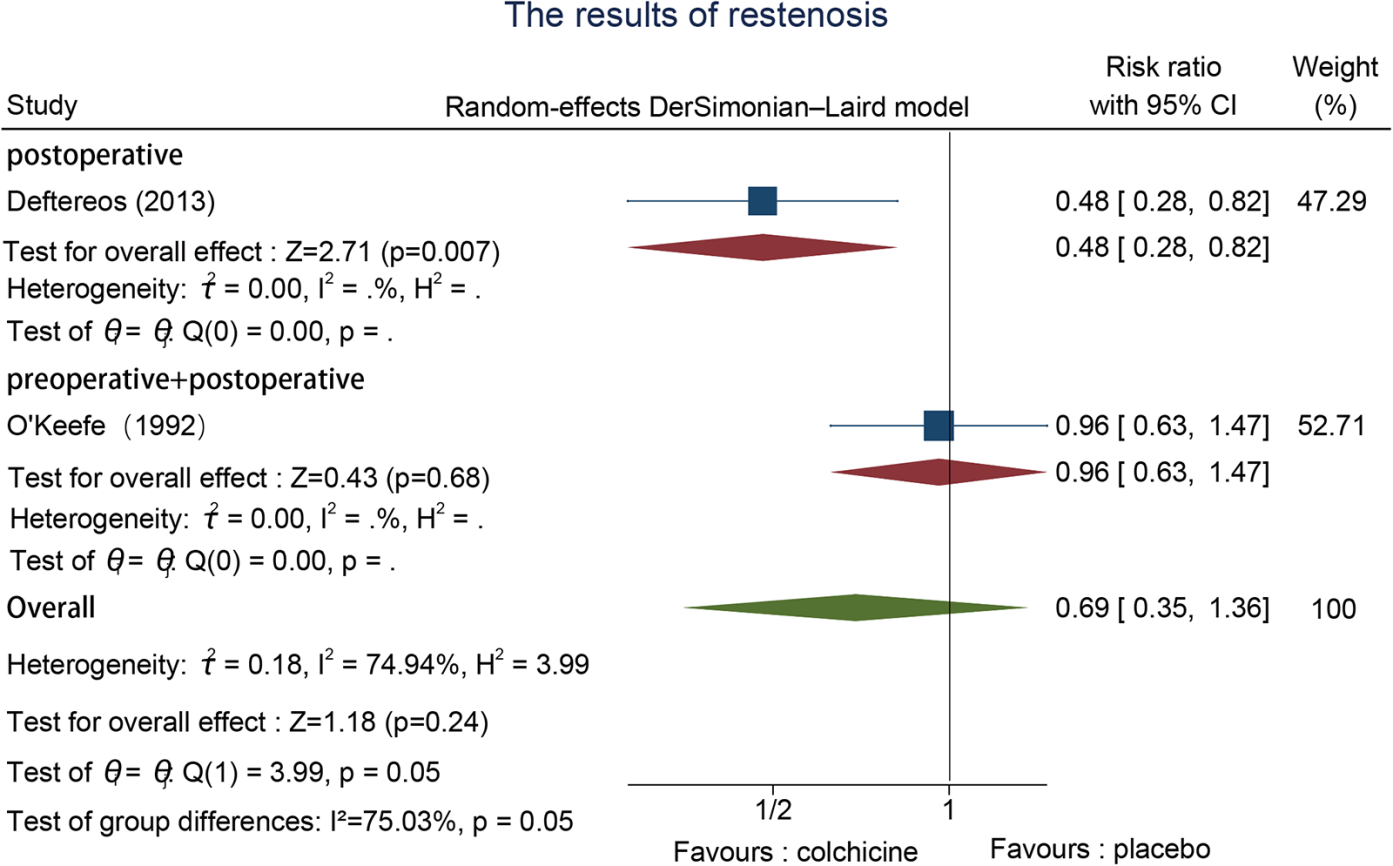
The results of restenosis.

A total of two studies reported ISR events, one postoperative (22) and one pre-and postoperative (31). A random-effects model was selected to assess the significant data heterogeneity we found in two trials that reported restenosis. The effectiveness of colchicine use for ISR was not statistically significant in contrast to the control group (RR = 0.69, 95% CI [0.35, 1.36], *p* = 0.24) (Figure 10).

#### 3.6.9. Subgroup analysis of different doses and dosing times

There were no considerable differences in dose size and treatment duration between the two preoperative studies, but there were differences in dose administration and treatment duration in the postoperative group and the pre-and postoperative group, so we performed subgroup analyses for various dose administration and treatment duration depending on the time point. In the postoperative group, the daily dose varied from 0.5 mg to 1 mg, and the treatment period lasted from 1 month to 19.6months; while the pre-and postoperative group's daily doses spanned between 0.5 mg to 2 mg, and the duration of the treatment period lasted from five days to twelve months. Subgroup analysis of various doses and durations of administration for hs-CRP, IL-6, stroke, and stent thrombosis revealed no significant differences. The administration of 1 mg was slightly more effective than 0.5 mg in the postoperative group, according to a subgroup analysis of MACE, although the heterogeneity between groups was not significant (*I*^2 ^=^ ^29.7%, *p* = 0.24); however, a subgroup analysis of adverse events revealed that, in the presence of adverse events, the impact of 0.5 mg in the postoperative intervention group was remarkably less than that of 1 mg, heterogeneity between groups (*I*^2 ^= 70%, *p* = 0.03) (Supplementary Tables S1–S4).

#### 3.6.10. Heterogeneity discussion

**Figure 11:**
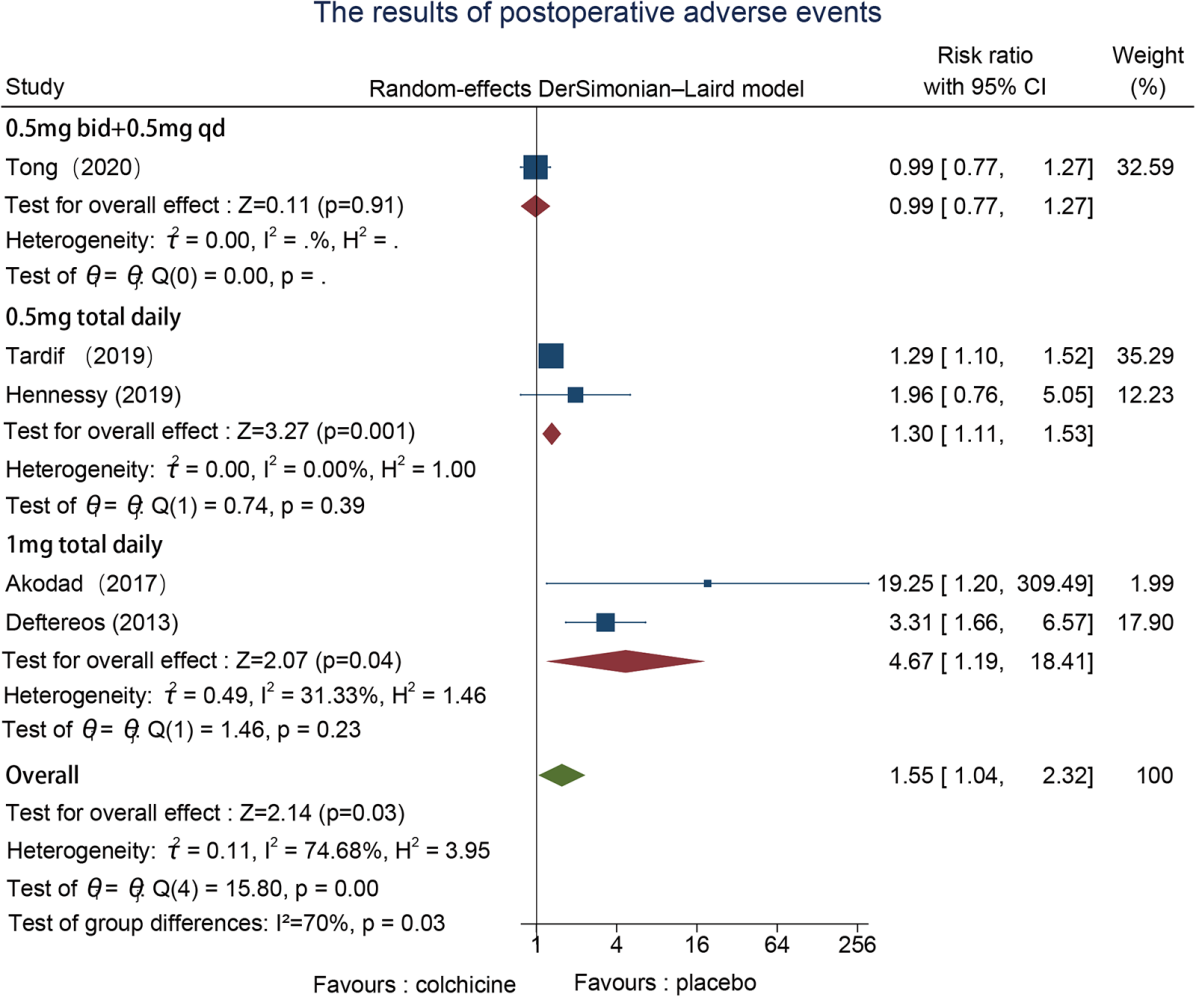
Subgroup analyses of postoperative adverse events according to colchicine dose.

Nine indicators overall were used in this systematic evaluation. The results revealed significant heterogeneity in the assessment of adverse events, therefore we discussed the causes of heterogeneity. For the analysis of adverse events, we found that the heterogeneity mainly derived from the postoperative group, so we performed subgroup analysis by dose and duration of treatment, and the results showed greater heterogeneity between groups with different doses (*I*^2^ = 70%, *p* = 0.03), therefore, we suggest that the source of this indicator heterogeneity may be due to differences in dose (Figure 11).

#### 3.6.11. Publication bias

**Figure 12:**
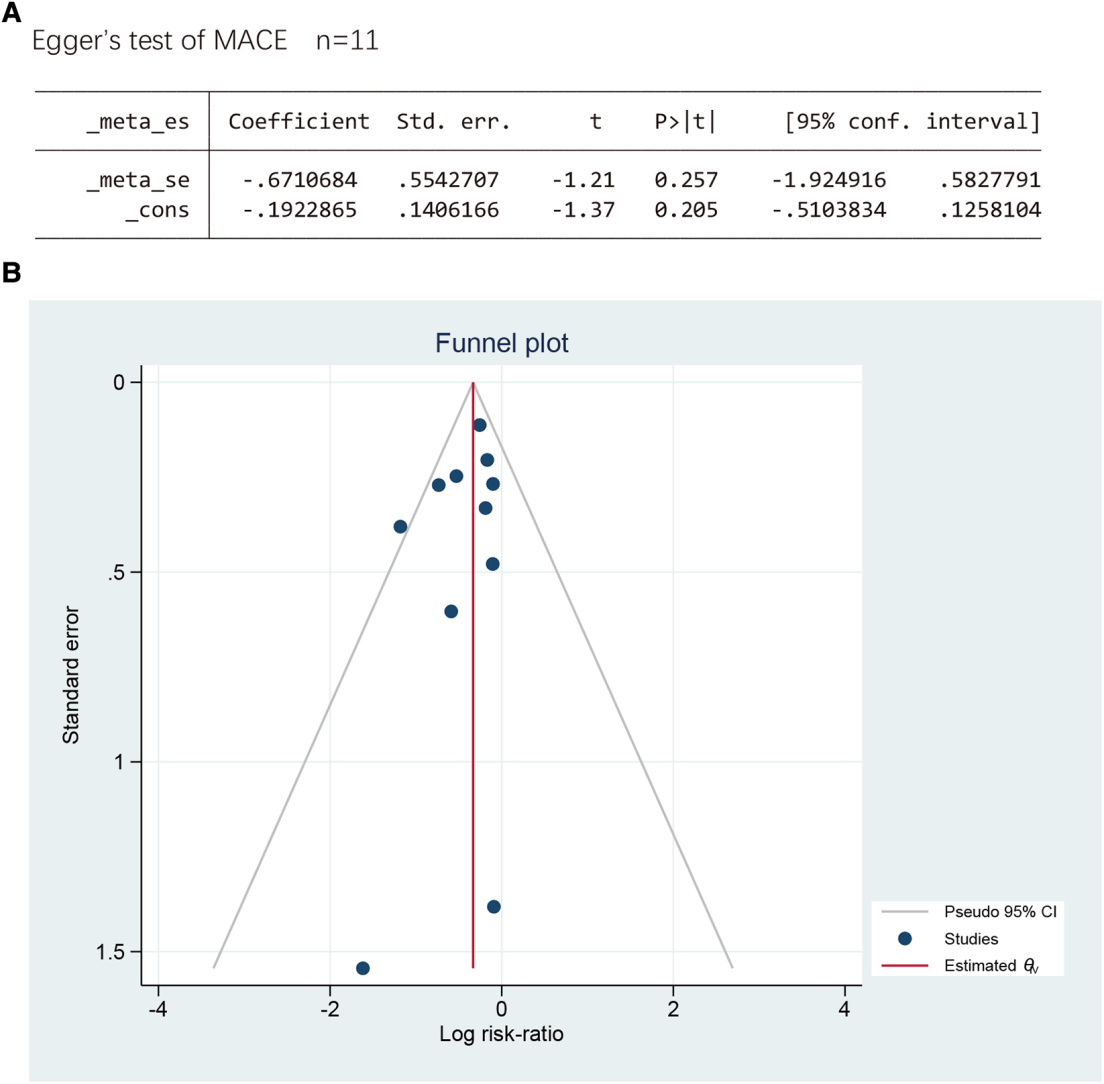
(**A**) The Egger’s test. (**B**) The funnel plot.

Since ten studies have reported MACEs, we verified publication bias by funnel plot and Egger's test. The outcomes revealed respectable *P* > |*t*| = 0.257, suggesting low publication bias (Figure 12).

## 4. Discussion

### 4.1. Overview

Overall 12 RCTs were selected for this systematic evaluation, including two preoperative groups, five postoperative groups, and five pre-and postoperative groups to assess the efficacy of colchicine at the various time points in the perioperative period of PCI for CAD. The outcomes revealed that colchicine was effective in preventing postoperative stroke events, reducing the formation of stent thrombosis, and decreasing the level of postoperative inflammation compared with the control group, suggesting that colchicine may reduce the instability of coronary atherosclerotic plaques and thus the formation of stent thrombosis by decreasing the level of postoperative inflammation, thus reducing the occurrence of cerebrovascular events; however, the effects of mortality, ISR, and MI were not statistically significant.

### 4.2. Comparison with previous systematic reviews and new findings

In the peri-procedural period of PCI, our study concluded that the statistics of all-cause mortality events, ISR, and MI were not meaningful, However, they were relevant in reducing the occurrence of post-procedural MACE and preventing in-stent thrombosis, which accords with the conclusions of several recent systematic reviews (33–35). In addition, in a previous meta-analysis of colchicine in CAD, colchicine was useful in avoiding stroke in CAD patients, as demonstrated by a systematic study (36); this study (37) supports the administration of low doses to decrease the occurrence of postoperative adverse events; colchicine decrease the risk of associated cardiovascular events by reducing levels of inflammatory factors, and both studies are consistent with our opinion (38, 39); however, this study (40) found colchicine to be effective in preventing recurrent myocardial infarction, which may be related to the fact that t PCI was not used to treat the bulk of the study's experimental group. Nevertheless, none of the previous research assessed the effectiveness of colchicine at various intervals during the PCI perioperative period.

Supported by the findings of our meta-analysis, colchicine markedly raised the experimental group's risk of non-cardiovascular events (gastrointestinal symptoms, allergic reactions, etc.), and it was also more effective than the control group at preventing post-PCI stroke, in-stent thrombosis, and reducing post-operative hs-CRP and IL-6 levels. However, in contrast to previous studies, our study of colchicine focuses on the diverse effects of preoperative and postoperative administration at various time points to identify the ideal time point for the administration.

Subgroup analysis showed that the differences in the effect of different time points and total daily doses in reducing the occurrence of postoperative MACE were not statistically significant, while the relative risk of side effects was lesser in the low-dose group vs. the high-dose group, indicating that we may be able to choose low-dose continuous dosing to obtain the same effect while reducing the pain caused by side effects of the drug. In contrast, although the pre-and postoperative groups had a higher effect on reducing hs-CRP levels than the other two groups, we think that this may be because the high dose of colchicine given preoperatively led to an earlier increase in plasma concentration, which allowed colchicine to act more rapidly and effectively even though it caused an increase in inflammation after PCI. At the same time, the effect in the postoperative group was slightly lower than in the pre-and postoperative groups but much longer than in the other intervention groups, perhaps implying that continued long-term administration is also a key factor in reducing inflammation levels, so we suggest that for reducing postoperative inflammation levels, preoperative administration and continued postoperative administration for at least one month is the best way to use the drug, rather than limiting it to a single time point in the perioperative period. Prevention of stroke events is more effective when administered postoperatively; the effect of all-cause mortality was not statistically significant and did not differ significantly between subgroups, indicating that colchicine has certain safety and is not influenced by the time point of administration; although our study does not support the effectiveness of colchicine in preventing postoperative restenosis, we found significant heterogeneity among this subgroup, which may be related to the fact that O'Keefe's experimental population included patients undergoing balloon angioplasty without stent implantation, that the mechanism of stenosis after coronary balloon dilation is different from that of in-stent restenosis, and that the anti-inflammatory effect of colchicine may make it more suitable for PCI stenting (41, 42). In addition, we found inconsistencies between the two preoperative studies about the efficacy of colchicine in decreasing postoperative myocardial injury after PCI, which may be a promising area for further study.

Within the 12 studies included, encompassing patients with both acute coronary syndrome and chronic coronary syndrome, each study recruited patients based on different criteria and defined different endpoints. However, in the several cardiovascular outcomes analyzed, no significant heterogeneity was observed; postoperative adverse events were the single outcome with the highest heterogeneity in our study, but in subgroup analyses we found that the source of heterogeneity was due to the drug dose, which may mean that the acuity of coronary artery disease in patients did not influence the secondary prevention of CAD by colchicine. In our study, colchicine demonstrated a significant reduction in the occurrence risk of the primary composite outcome, MACE, this reduction was primarily driven by a 56% decrease in the risk of postoperative stroke events and a 51% decrease in the risk of in-stent thrombosis events; post-angioplasty restenosis, driven predominantly by arterial elastic recoil and remodeling, and ISR, primarily attributed to neointimal hyperplasia and localized inflammation (16), pose concerns in our study, upon analyzing the experimental results, it appears that colchicine may be more suitable for the treatment of ISR; the inflammatory response during the perioperative period is closely associated with MACE, and the anti-inflammatory effects of colchicine have been validated in our study, however, in two studies involving preoperative administration, the elevation of hs-CRP and IL-6 was obviously suppressed, showing no significant difference compared to the control group. Nevertheless, considering the prominent reduction in perioperative myocardial infarction risk with high-dose statin therapy administered preoperatively, the combination of colchicine may be an effective treatment strategy for preventing post-PCI MACE (43).

### 4.3. Anti-inflammatory mechanisms and side effects

Colchicine's anti-inflammatory action results from a variety of mixture effects. In the background of coronary atherosclerosis, the inflammatory endothelium will continuously attract migration, adhesion, and activation of leukocytes, which subsequently activate the release of neutrophil granulocyte enzymes, allowing increased instability of intravascular plaques (44), there may be a correlation between the extent of the thrombus creation and further stimulation of thrombin formation and promotion of fibrin production while restoring factor Xa activity (45–47); due to the lack of P-Glycoprotein transport, which makes colchicine more inclined to accumulate in neutrophil and affect their activity (16, 48);At the same time, it inhibits the directional migration of neutrophils to inflammatory lesions, and reduces the adhesion of neutrophils to inflammatory endothelial cells by reducing the quantitative expression of L-selectin adhesion molecules and the qualitative expression of E-selectin adhesion molecules (49). Our statistical findings revealed that colchicine could reduce the incidence of in-stent thrombosis by 51%, although it was temporarily impossible to assess whether it was related to the time point of administration, at least partly due to its anti-inflammatory effect.

Although the specific mechanism is not clear at present, existing research has demonstrated that one of the crucial aspects of aseptic inflammation is the activation of the NLRP3 inflammasome, and the subsequent release of interleukin (IL)-1β will lead to vascular inflammation. Therefore, blocking the assembly and activation of NLRP3 inflammasome seems to be a new target for the treatment of cardiovascular diseases (50). Colchicine inhibits the activation of NLRP3 inflammasome and thus reduces the production of interleukin (IL)-1β and IL-18 mediated by it (51, 52) since neutrophil enzymes activate IL-1β and IL-18 extracellularly, and colchicine also inhibits the reduction of the release of neutrophil granulocyte enzymes associated with thrombosis, which is one of its potential anti-inflammatory mechanisms (53, 54). Finally, these compounding effects will result in an overall decrease in IL-6 production and hs-CRP concentrations. Our meta-analysis suggests that the acute preoperative administration of colchicine at higher doses and long-term postoperative administration is more effective in reducing IL-6 and hs-CRP levels and that the reduction in inflammation levels is a key pathological basis for the reduction of all types of adverse cardiovascular events.

In addition, circulating monocyte-platelet aggregation (MPA), one of the markers of acute myocardial infarction, colchicine activates neutrophils to release neutrophil extracellular traps, externalized nucleosomes and chromatin-adherent neutrophil enzymes that accumulate in rupture-prone plaques, thereby interfering with the interaction between platelets and leukocytes (55, 56).

As the only non-targeted anti-inflammatory drug available, colchicine also has good safety (57), as evidenced by our studies. Although there is a possible immunosuppressive effect, in a postoperative study (27) we found an increased incidence of sepsis as a possible cause of their non-cardiovascular mortality, and this increased incidence was found at follow-up after discontinuation of the drug, which is a matter of concern; at the same time, the high-dose administration of this study in the first month after surgery may also be one of the potential factors. In addition, the LoDoCo2 trial (58) also revealed that the colchicine group had considerably more non-cardiovascular mortality than the placebo group, while the incidence of new tumors and hospitalizations for infections was similar between the two groups, suggesting that we cannot simply attribute this alone. Therefore, the dosage of colchicine is still a problem to consider. Our study also demonstrated that postoperative adverse events, such as gastrointestinal syndrome and allergic reactions, were related to the use of colchicine. In the subgroup analysis of various doses, it revealed that this connection was derived from the use of dose, which means that we may be able to prevent it by using low-dose administration regimens, however, it is unclear whether the relatively positive therapeutic effect of colchicine on cardiovascular results outweighed the potential adverse effects on non-cardiovascular outcomes.

### 4.4. Research limitations and implications

First of all, with the restricted amount of original literature, especially in the preoperative study, only two items were included and more attention was paid to the measurement of inflammatory response indicators. The postoperative and preoperative and postoperative groups mainly focused on stoke and stent thrombosis, but the quantitative analysis results cannot be obtained from IL-6. When more randomized controlled trials appear, we will update the systematic review; Second, since different degrees of risk bias existed in the enrolled research, we consider that colchicine treatment-related coronary artery disease should be conducted in compliance with the CONSORT guidelines to improve the quality of randomized controlled trials (59); Third, because this original literature involves many countries and the diagnostic criteria of coronary artery disease in different countries may have some differences, which may limit our results; Fourth, although there were a few patients in the three trials (26–28) that were included who did not receive PCI treatment and only got pharmacological therapy, the absolute number of these patients was relatively small, thus their absence had minimal impact on the study's overall conclusion. At present, we have noticed that more and more randomized trials have begun to evaluate the different effects of colchicine on coronary artery disease. As a drug with great potential in the cardiovascular field, we would like to see more multi-center, large sample randomized controlled trials to find the optimal timing of colchicine use.

## 5. Conclusion

When performing perioperative PCI for CAD, colchicine is efficient at all phases. For postoperative dosing, we advise a total daily dose of 0.5 mg of continuous dosing for reducing the occurrence of MACE, which not only decreases the financial burden on the patient but also minimizes the side effects of the drug and achieves the same effect as the drug; postoperative drug administration is more effective in preventing postoperative stroke events, but the optimal time to decrease the level of inflammatory response is not during the perioperative phase, to obtain the highest benefits, we recommend that drugs are used before and after surgery for at least one month. However, the evaluated studies' relatively small sample sizes indicate they are not high quality. Therefore, large, multicenter randomized controlled studies are still required to support our conclusions.

## Data Availability

The original contributions presented in the study are included in the article/**Supplementary Material**, further inquiries can be directed to the corresponding author.
