# Supplementary material for: Colchicine efficacy comparison at varying time points in the peri-operative period for coronary artery disease: a systematic review and meta-analysis of randomized controlled trials
Source: Front Cardiovasc Med. 2023 Aug 4;10:1156980. doi: 10.3389/fcvm.2023.1156980 (PMC10438985; doi:10.3389/fcvm.2023.1156980)
Supplement: Supplementary file 1 [file Table1.docx]

**Supplementary text 1.** The details of search terms and strategies. The searching methods for the included articles in the ﻿databases are almost the same. Take searching Pubmed as an example, the search terms and strategies are as follows:

#1 ﻿Colchicine [MeSH Terms]

#2 Colchicine [Title/Abstract]

#3 #1 OR #2

#4 Coronary artery disease [MeSH Terms]

#5 Coronary artery disease [Title/Abstract]

#6 Left main coronary artery disease [MeSH Terms]

#7 Left main coronary artery disease [Title/Abstract]

#8 Coronary arteriosclerosis [MeSH Terms]

#9 Coronary arteriosclerosis [Title/Abstract]

#10 #4 OR #5 OR #6 OR #7 OR #8 OR #9

#11 Acute coronary syndrome [MeSH Terms]

#12 Acute coronary syndrome [Title/Abstract]

#13 Coronary syndrome [MeSH Terms]

#14 Coronary syndrome [Title/Abstract]

#15 Acute coronary [MeSH Terms]

#16 Acute coronary [Title/Abstract]

#17 #11 OR #12 OR #13 OR #14 OR #15 OR #16

#18 Percutaneous coronary intervention [MeSH Terms]

#19 Percutaneous coronary intervention [Title/Abstract]

#20 Coronary intervention [MeSH Terms]

#21 Coronary intervention [Title/Abstract]

#22 Percutaneous coronary [MeSH Terms]

#23 Percutaneous coronary [Title/Abstract]

#24 Percutaneous coronary revascularization [MeSH Terms]

#25 Percutaneous coronary revascularization [Title/Abstract]

#26 Coronary revascularizations [MeSH Terms]

#27 Coronary revascularizations [Title/Abstract]

#28 #18 OR #19 OR #20 OR #21 OR #22 OR #23 OR #24 OR #25 OR #26 OR #27

#29 Randomized Controlled Trial [Publication Type]

#30 Controlled Clinical Trial [Publication Type]

#31 Random [All Fields]

#32 #29 OR #30 OR #31

#33 #3 AND #10 AND #17 AND #28 AND #32

**Supplementary Table S1. Subgroup analyses of postoperative trials according to colchicine dose**

| Outcomes | Number of studies | Effect measures | 95%CI | p-value | I^2^（%） | Heterogeneity between groups |
| --- | --- | --- | --- | --- | --- | --- |
| Major cardiovascular events |  |  |  |  |  |  |
| 0.5mg bid+0.5mg qd | 1 | RR: 0.59 | 0.36-0.96 | 0.03 | 0 |  |
| 0.5mg total daily | 2 | RR: 0.77 | 0.61-0.95 | 0.02 | 0 |  |
| 1mg total daily | 2 | RR: 0.50 | 0.29-0.83 | 0.008 | 0 | P=0.24 |
| Stroke |  |  |  |  |  |  |
| 0.5mg bid+0.5mg qd | 1 | RR: 0.34 | 0.07-1.65 | 0.18 | 0 |  |
| 0.5mg total daily | 1 | RR: 0.26 | 0.10-0.71 | 0.008 | 0 |  |
| 1mg total daily | 1 | RR: 2.88 | 0.12-69.87 | 0.52 | 0 | P=0.37 |
| Stent thrombosis |  |  |  |  |  |  |
| 0.5mg bid+0.5mg qd | 1 | RR: 0.50 | 0.25-1.02 | 0.06 | 0 |  |
| 0.5mg qd | 1 | RR: 0.33 | 0.01-8.03 | 0.50 | 0 | P=0.80 |
| Adverse events |  |  |  |  |  |  |
| 0.5mg bid+0.5mg qd | 1 | RR: 0.99 | 0.77-1.27 | 0.91 | 0 |  |
| 0.5mg total daily | 2 | RR: 1.30 | 1.11-1.53 | 0.001 | 0 |  |
| 1mg total daily | 2 | RR: 4.67 | 1.19-18.41 | 0.03 | 31.3 | P=0.03 |

Note: qd, once a day; bid, twice a day. 0.5mg bid+0.5mg qd：during the first month, 0.5mg bid, and for the next eleven months, 0.5mg qd

**Supplementary Table S2. Subgroup analyses of postoperative trials according to Dosing time**

| Outcomes | Number of studies | Effect measures | 95%CI | p-value | I^2^（%） | Heterogeneity between groups |
| --- | --- | --- | --- | --- | --- | --- |
| Major cardiovascular events |  |  |  |  |  |  |
| <1 month | 2 | RR: 0.46 | 0.06-3.48 | 0.45 | 0 |  |
| >1 month | 3 | RR: 0.66 | 0.49-0.87 | 0.003 | 38 | P=0.74 |
| Stent thrombosis |  |  |  |  |  |  |
| <1 month | 1 | RR: 0.33 | 0.01-8.03 | 0.68 | 0 |  |
| >1 month | 1 | RR: 0.50 | 0.25-1.02 | 0.06 | 0 | P=0.80 |
| hs-CRP |  |  |  |  |  |  |
| <1 month | 1 | SMD:-0.17 | -0.43，0.09 | 0.21 | 0 |  |
| >1 month | 1 | SMD:-0.37 | -0.65，-0.10 | 0.01 | 0 | P=0.37 |
| Adverse events |  |  |  |  |  |  |
| <1 month | 2 | RR: 4.42 | 0.43，45.10 | 0.21 | 63 |  |
| >1 month | 3 | RR: 1.40 | 0.93，2.10 | 0.10 | 82 | P=0.34 |

**Supplementary Table S3. Subgroup analyses of preoperative and postoperative trials according to colchicine dose**

| Outcomes | Number of studies | Effect measures | 95%CI | p-value | I^2^（%） | Heterogeneity between groups |
| --- | --- | --- | --- | --- | --- | --- |
| Major cardiovascular events |  |  |  |  |  |  |
| 2mg total daily +0.5mg bid | 2 | RR: 0.75 | 0.36-1.56 | 0.44 | 0 |  |
| 1mg total daily +0.5mg qd | 1 | RR;0.83 | 0.43-1.59 | 0.57 | 0 |  |
| 0.5mg qd | 1 | RR: 0.31 | 0.15-0.65 | 0.002 | 0 |  |
| 0.6mg bid | 1 | RR: 0.85 | 0.57-1.26 | 0.41 | 0 | P=0.12 |
| Adverse events |  |  |  |  |  |  |
| 2mg total daily +0.5mg bid | 1 | RR: 2.71 | 0.11-65.60 | 0.54 | 0 |  |
| 0.6mg bid | 1 | RR: 2.46 | 1.28-4.74 | 0.007 | 0 | P=0.95 |

Note: qd, once a day; bid, twice a day. 2mg total daily +0.5mg bid: 2 mg dose preoperatively and 0.5 mg bid postoperatively；1mg total daily +0.5mg qd: 1 mg dose preoperatively and 0.5 mg qd postoperatively

**Supplementary Table S4. Subgroup analyses of preoperative and postoperative trials according to Dosing time**

| Outcomes | Number of studies | Effect measures | 95%CI | p-value | I^2^（%） | Heterogeneity between groups |
| --- | --- | --- | --- | --- | --- | --- |
| Major cardiovascular events |  |  |  |  |  |  |
| <1 month | 2 | RR: 0.75 | 0.36-1.56 | 0.44 | 0 |  |
| >1 month | 3 | RR: 0.66 | 0.48-0.89 | 0.008 | 67 | P=0.76 |
| Adverse events |  |  |  |  |  |  |
| <1 month | 1 | RR: 2.71 | 0.11-65.60 | 0.54 | 0 |  |
| >1 month | 1 | RR: 2.46 | 1.28-4.74 | 0.007 | 0 | P=0.95 |
