# Supplementary material for: Colchicine efficacy comparison at varying time points in the peri-operative period for coronary artery disease: a systematic review and meta-analysis of randomized controlled trials
Source: Front Cardiovasc Med. 2023 Aug 4;10:1156980. doi: 10.3389/fcvm.2023.1156980 (PMC10438985; doi:10.3389/fcvm.2023.1156980)
Supplement: Supplementary file 2 [file Table2.docx]

| **Study**  **(author/**  **year)** | **Key inclusion criteria** | **Key exclusion criteria** | **Time of colchicine**  **initiation and duration** | **Dosage** |
| --- | --- | --- | --- | --- |
| Shah et al. (2020) | suspected ischemic heart disease or acute coronary syndromes referred for clinically indicated coronary angiography with possible PCI | glomerular filtration rate <30 mL/min or on dialysis and intolerance to colchicine | First dose 1-2 hours before coronary angiography, second dose one hour later | 1.2 mg 1 to 2 hours before coronary angiography, followed by 0.6 mg 1 hour later |
| cole et al.（2021） | patients were included if they had a de-novo lesion amenable to PCI, and high-sensitive troponin-I and CK  had peaked and stabilized | Patients were excluded if they had active inflammation/infection;  had prior ACS within 12months;  had severe renal impairment | Medication given 6-24 hours before PCI procedure | 1 mg followed by 0.5 mg one hour later |
| Akodad et al.（2017） | The patient with ST segment elevation myocardial infarction underwent successful primary percutaneous coronary intervention | cardiogenic shock, severe chronic kidney failure ,colchicine intolerance or contraindication | colchicine was administered on the first day of the surgery and for 1 month, without a loading dose | 1 mg once daily |
| Tardif et al.（2019） | Patients who had an MI within  30 days before enrolment and  had completed any planned  percutaneous revascularisation  procedures | Patients if had stroke within previous 3 months, type two index MI, recent or planned CABG; inflammatory bowel disease or chronic diarrhea and severe renal disease | Once the patient grouping is completed, medication administration begins, with a median treatment duration of 22.6 months | 0.5 mg once daily |
| Tong et al.（2020） | ACS with presence of coronary disease | Requiring bypass surgery; severe liver impairment; severe renal impairment  (eGFR <30 mL/min/1.73 m^2^) | Patients start taking medication immediately after being assigned to a group, and the treatment duration is 12 months | 0.5mg oral colchicine twice daily for the first month, followed by 0.5mg daily for eleven months |
| Deftereos et al. (2013) | Diabetes and undergoing percutaneous coronary revascularization | Acute myocardial infarction; renal impairment (eGFR <20 mL/min/1.73 m^2^);liver failure | The patient began taking the medication on the day after PCI and continued for 6 months | 0.5 mg twice daily |
| Hennessy et al. (2019) | Adult patients were eligible for enrolment if they had sustained a type 1 acute MI | severe renal impairment; severe hepatic dysfunction; females of child-bearing age who are pregnant, lactating | Dosing started after completion of grouping and continued for one month | 0.5 mg once daily |
| O'Keefe et al.（1992） | Patients(CCS) who had undergone  successful coronary angioplasty | premenopausal women；active peptic ulcer disease and diarrhea；creatinine ≥2.5 mg/dI at baseline；known colchicine intolerance | The first dose should be administered within 24 hours before or after the surgery, and the treatment should continue for a duration of six months | 0.6 mg twice daily |
| Mewton et al.（2021） | All adult patients with a first-time STEMI referred for primary or rescue PCI admitted to the participating centers were screened against eligibility criteria | hemodynamic instability; any obvious contraindication to cardiac magnetic resonance imaging; severe liver or known renal dysfunction as defined by a glomerular filtration rate ≤30 mL/min and chronic treatment with colchicine | The preoperative administration of a loading dose of PCI was followed by postoperative medication for a duration of five days | 2-mg oral loading dose, followed by 0.5 mg twice a day |
| Zarpelon et al. (2016) | indication for elective  myocardial revascularization surgery | severe liver disease and renal failure; known gastrointestinal diseases | Treatment group started preoperative dosing until discharge | 1mg given twice a day within 24 hours before surgery, 0.5mg twice a day after surgery |
| Akrami et al. (2021) | All the patients underwent coronary angiography and were managed with either PCI or medical therapy | any history of long-term colchicine use or hypersensitivity to it, moderate renal dysfunction (glomerular fltration rate˂50) | Dosing started after completion of grouping and continued for six months | 0.5 mg once daily |
| Hosseini et al, (2022) | The patient was diagnosed with acute STEMI and underwent PCI within 12 hours | cardiogenic shock; colchicine  intolerance; renal failure (estimated glomerular filtration rate,30 mL/min) | administered immediately before PCI in patients with STEMI; continuous postoperative dosing for one year | 1mg given preoperatively, followed by another 0.5 mg administered  orally each day |

Note: CK, creatinine kinase; eGFR, estimated glomerular filtration rate; PCI, percutaneous coronary intervention; ACS, acute coronary syndrome; CCS, chronic coronary syndrome; MI, myocardial infarction; STEMI, ST-segment elevation myocardial infarction; CABG, coronary artery bypass graft.
