# Supplementary material for: Colchicine efficacy comparison at varying time points in the peri-operative period for coronary artery disease: a systematic review and meta-analysis of randomized controlled trials
Source: Front Cardiovasc Med. 2023 Aug 4;10:1156980. doi: 10.3389/fcvm.2023.1156980 (PMC10438985; doi:10.3389/fcvm.2023.1156980)
Supplement: Supplementary file 3 [file Table3.docx]

| **Study**  **(author/**  **year)** | **Sample size**  **(T/C)** | **Age (years)**  **(T/C)** | **Male (%)** | **Drug**  **intervention**  **time** | **Dosing**  **time** | **Intervention**  **(T/C)** | | **Main**  **outcomes** | | **PCI (%)** |
| --- | --- | --- | --- | --- | --- | --- | --- | --- | --- | --- |
| Shah et al. (2020) | 400 (206/194） | 59/62 | 93.5 | preoperative | 2 hours | colchicine（1.2mg+0.6 mg） | placebo | | ①②④⑤⑥⑦⑧ | 100 |
| cole et al.（2021） | 75 (36/39) | 63.7±6.9/63.5±7.2 | 72.0 | preoperative | 6-24 hours | colchicine（1mg+0.5mg） | placebo | | ④ | 100 |
| Akodad et al.（2017） | 44 (23/21) | 60.1±13.1/59.7±11.4 | 79.5 | postoperative | 1 month | colchicine  （1mg qd） | placebo | | ①⑥⑧ | 100 |
| Tardif et al.（2019） | 4745 (2366/2379） | 60.6±10.7/60.6±10.7 | 80.8 | postoperative | 22.6 months | colchicine  （0.5mg qd） | placebo | | ①②④⑥⑦ ⑧ | 92.9 |
| Tong et al.（2020） | 795 (396/399） | 59.7±10.2/60.0±10.4 | 79.4 | postoperative | 12 months | colchicine（0.5mg bid+0.5mg qd） | placebo | | ①②③⑥⑦ | 86.9 |
| Deftereos et al. (2013) | 196  (100/96) | 63.7±6.9/63.5±7.2 | 65.3 | postoperative | 6 months | colchicine  （0.5mg bid） | placebo | | ①②⑥⑦⑨ | 100 |
| Hennessy et al. (2019) | 237 (119/118) | 61±13.6/ 61±12.5 | 76.7 | postoperative | 1 month | Colchicine  （0.5mg qd） | placebo | | ①③④⑤⑥⑧ | 100 |
| O'Keefe et al.（1992） | 197  (130/67) | 59/62 | 85.7 | preoperative postoperative | 6 months | Colchicine  （0.6mg bid） | placebo | | ①⑥⑦⑨ | 100 |
| Mewton et al.（2021） | 192  （101/91） | 59.0±10.6/60.9±10.4 | 80.2 | preoperative postoperative | 5 days | colchicine（2mg+0.5mg bid） | placebo | | ①②④⑥ | 100 |
| Zarpelon et al. (2016) | 140  (71/69) | 61.5±10.3/60.3±8.1 | 67.8 | preoperative postoperative | <1month | colchicine（1mg bid+0.5mg bid） | placebo | | ①⑦ | 100 |
| Akrami et al. (2021) | 249 (120/129) | 56.9±7.56/56.89±7.45 | 69.4 | preoperative postoperative | 6 months | Colchicine  （0.5mg qd） | placebo | | ①⑦ | 86.3 |
| [Hosseini et al, （2022）](#_ENREF_24) | 321  (120/129) | 58.7±10.4/58.9±11.2 | 79.1 | preoperative postoperative | 12 months | colchicine（1mg +0.5mg qd） | Placebo | | ① | 100 |

Note: T, trial group; C, control group; qd, once a day; bid, twice a day. ①MACEs; ②stroke; ③stent thrombosis; ④hs-CRP; ⑤IL-6; ⑥adverse events；⑦mortality; ⑧MI; ⑨ISR
